# Toward the Association Between EFL/ESL Teachers' Work Engagement and Their Students' Academic Engagement

**DOI:** 10.3389/fpsyg.2021.739827

**Published:** 2021-08-17

**Authors:** Aizhen Zhang, Yang Yang

**Affiliations:** ^1^School of International Studies, Henan Finance University, Zhengzhou, China; ^2^School of Education, Henan Finance University, Zhengzhou, China

**Keywords:** teacher work engagement, student academic engagement, EFL/ESL teachers, learning, academic engagement

## Abstract

Given the fact that EFL/ESL students' academic engagement is of high importance for their learning success, numerous studies have been carried out to identify factors contributing to students' engagement. However, the role of teacher personal factors, notably teacher work engagement has received scant attention. Moreover, no review study has been conducted on this issue. Accordingly, the present review intends to explicate the multidimensional essence of teacher work engagement and student academic engagement and the association between these constructs. In light of the theoretical and empirical evidence, the role of EFL/ESL teachers' work engagement in improving their students' academic engagement was proved. The pedagogical implications of the findings are also highlighted.

## Introduction

Due the fact that students' academic engagement is at the heart of their success (Carver et al., 2021), it has received a remarkable attention in field of education, notably second language education. Student academic engagement refers to “the amount (quantity) and type (quality) of students' active participation and involvement in language learning tasks/activities” (Hiver et al., 2021b, p. 2). As put forward by Baralt et al. (2016), student academic engagement as a complex and multidimensional concept encompasses a range of factors that interact to demonstrate students' positive emotions toward the learning process. Regarding the importance of EFL/ESL students' academic engagement in their success, Hiver et al. (2021a) postulated that students' academic engagement is intertwined with their learning success, mainly due to the fact that students with a high level of emotional, behavioral, and cognitive engagement typically put more effort to learn the resources and materials. Hence, It is essential to investigate factors contributing to EFL/ESL students' academic engagement. Numerous research studies endeavored to investigate the probable role of students' personal factors in their academic engagement (e.g., Wang and Eccles, 2013; Kahu et al., 2015; Qureshi et al., 2016; Ramshe et al., 2019; Khajavy, 2021). Additionally, some previous studies have been conducted on teachers' interpersonal variables to examine their effects on students' learning engagement (e.g., Estepp and Roberts, 2015; Imlawi et al., 2015; Derakhshan, 2021; Xie and Derakhshan, 2021; Zheng, 2021). Nevertheless, a few studies have been carried out to probe the casual relationship between teachers' personal factors such as work engagement and students' academic engagement (e.g., Wu, 2010; Cardwell, 2011; Cinches et al., 2017).

Teacher work engagement as the prime instance of teacher personal factors refers to “a persistent, positive affective-motivational state of fulfillment that is characterized by vigor, dedication, and absorption dimensions” (Maslach et al., 2008, p. 104). In a more comprehensive definition, Cardwell (2011) characterized teachers' work engagement as their “interest in,” “enthusiasm for,” and “investment in” teaching (p. 17). To him, interested and enthusiastic teachers are able to effectively engage their students in the learning process. Similarly, Cinches et al. (2017) also suggested that highly engaged teachers have a sense of inspiration and enthusiasm that enable them to teach more effectively. They explained that one of the key factors contributing to students' academic engagement is the quality of instruction.

In spite of the fact that teachers' work engagement may affect their students' academic engagement (Cardwell, 2011; Hospel and Garland, 2016; Cinches et al., 2017), a limited number of studies have examined the relationship between these two variables. In addition, no review study has been conducted to illustrate teacher work engagement, student academic engagement and the association between these two variables. Hence, in this review study, the researcher attempted to explain the complex and multifaceted nature of teaching and learning engagement, on the one hand, and to illustrate the positive relationship between EFL/ESL teachers' work engagement and their students' academic engagement.

### Teacher Work Engagement

Teacher work engagement is conceptualized as “a positive, fulfilling, work-related state of mind,” comprising three dimensions of *absorption, dedication*, and *vigor* (Schaufeli et al., 2002, p. 75). Absorption is described by being deeply focused and joyfully immersed in one's vocation (Bakker et al., 2008). Dedication, as the second component of work engagement, is characterized by being totally engaged in one's profession, and having a sense of “significance, enthusiasm, inspiration, pride, and challenge” (Bakker et al., 2008). Finally, vigor is defined as having a great deal of energy while working, being inclined to put effort in one's vocation, and remaining persistent in challenging situations (Schaufeli et al., 2002). Taken together, engaged teachers are more concentrated on, dedicated to, and passionate about their profession (Hakanen et al., 2006).

### Student Academic Engagement

The notion of student academic engagement is conceptualized in different ways. That is, there has been a debate over the definition of this concept. Skinner et al. (2009), for instance, defined student academic engagement as “the quality of students' participation or connection with the educational endeavor and hence with activities, values, individuals, aims, and place that comprise it” (p. 495). Later, Philp and Duchesne (2016) defined students' engagement in terms of the quantity and quality of their effort in fulfilling their academic responsibilities.

Like its definition and conceptualization, there is a range of controversy regarding the dimensions and components of student academic engagement (Table 1). For instance, Schaufeli et al. (2002) proposed “*Vigor, Absorption*, and *Dedication*” as three components of student academic engagement, as opposed to Jimerson et al. (2003) who enumerated “*Academic Engagement, Behavioral Engagement*, and *Cognitive Engagement*” as the main dimensions of this concept. As another example, Finn (1989) divided students' academic engagement into two dimensions of “*Participation* and *Identification*,” whereas Willms (2003) classified the components of student academic engagement into two categories of “*Behavioral Engagement* and *Psychological Engagement*.”

**Table 1:** Components of student academic engagement.

**References**	**Components of student academic engagement**
Finn (1989)	▪ Participation: Students' active participation in classroom tasks/activities ▪ Identification: Students' sense of belongingness
Schaufeli et al. (2002)	▪ Vigor: Students' amount of effort and persistence in challenging situations ▪ Absorption: Students' immer sion in classroom tasks/activities ▪ Dedication: Students' sense of inspiration and enthusiasm toward the learning process
Jimerson et al. (2003)	▪ Affective Engagement: Students' emotions and attitudes toward teachers, classmates, and classroom context ▪ Behavioral Engagement: Students' observable actions ▪ Cognitive Engagement: Students' viewpoints about themselves, teachers, classmates, and instructional-learning context
Willms (2003)	▪ Behavioral Engagement: Students' participation in academic/non-academic activities ▪ Psychological Engagement: Students' sense of belongingness/attachment

Among the aforementioned models of student academic engagement, the model of Schaufeli et al. (2002) has been more prevalent. That is, several empirical and theoretical studies (e.g., Alrashidi et al., 2016; Morales-Rodríguez et al., 2019; Derakhshan, 2021) employed this model to illustrate the multidimensional nature of student academic engagement.

### The Positive Relationship Between EFL/ESL Teachers' Work Engagement and Their Students' Academic Engagement

Concerning the importance of English language teachers' work engagement in their students' academic engagement, Van Uden et al. (2013) suggested that those teachers' who are interested in and enthusiastic about their vocation foster their students' engagement. To them, engaged teachers can easily shape the classroom atmosphere in a way that students enjoy the learning process. This, in turn, encourages students to actively participate in classroom tasks and activities. Similarly, Hospel and Garland (2016) also stated that teachers' level of engagement can make a huge difference to students' sense of inspiration, commitment, and enthusiasm toward the learning process. They explained that engaged teachers are able to provide a stimulating learning environment wherein students' tendency to become involved in the learning process can be dramatically enhanced. Additionally, Taylor and Parsons (2011) proposed that highly engaged teachers commonly put more effort to teach the materials effectively. To them, when students witness teachers striving to teach them effectively, they will be motivated to take part in classroom activities.

## Empirical Studies

For more than three decades, the primary concerns of research in the domain of language education were teacher and students' negative variables such as burnout, disengagement, and dropout (e.g., Cephe, 2010; Mukundan and Khandehroo, 2010; Jahedizadeh et al., 2016; Seifalian and Derakhshan, 2018; Fathi et al., 2021). However, in recent years, interest in the school of “*Positive Psychology*” has inspired researchers to turn their focus to more positive variables. Accordingly, attention shifted from dropout and burnout to academic engagement and work engagement, respectively. In this regard, several scholars attempted to investigate teacher work engagement, student academic engagement, and their educational consequences. However, a limited number of studies have been conducted on the association between these two variables (e.g., Wu, 2010; Cardwell, 2011; Cinches et al., 2017). Cardwell (2011), for instance, tried to explore the association between teacher work engagement and student academic engagement. Employing self-report and observer-report questionnaires, participants' viewpoints toward the role of teacher work engagement in student academic engagement were gathered. Based on the results of analyses, the researcher submitted that teachers' work engagement can positively influence their students' academic engagement. In a similar vein, Cinches et al. (2017) also endeavored to examine the probable relationship between teacher effectiveness, teacher engagement, and student academic engagement. To do so, three pre-developed scales were distributed among the participants (i.e., 2,238 students, 98 teachers). Analyzing respondents' answers to the questionnaires revealed that both teacher effectiveness and engagement can positively predict student engagement.

## Conclusion and Pedagogical Implications

In the current review, two important concepts of teacher work engagement and student academic engagement, their definitions, and their underlying components were fully illustrated. Further, using theoretical and empirical evidence, the favorable association between these two variables was explained. In light of the existing evidence, it can be concluded that teachers' work engagement is critical in improving students' level of academic engagement. This finding appears to be beneficial for EFL/ESL teachers. Given the fact teachers' work engagement can positively predict their students' academic engagement (Cardwell, 2011; Hospel and Garland, 2016), those EFL/ESL teachers who intend to improve the academic engagement of their learners may enhance their professional engagement (i.e., vigor, absorption, dedication). It means that instead of pushing their students to participate in classroom activities, they should work on their own engagement in order to motivate students to become involved in the learning process.

## Author Contributions

AZ and YY read the relevant literature and explicated the multidimensional essence of teacher work engagement and student academic engagement and the association between these constructs. Both authors contributed to the article and approved the submitted version.

## Conflict of Interest

The authors declare that the research was conducted in the absence of any commercial or financial relationships that could be construed as a potential conflict of interest.

## Publisher's Note

All claims expressed in this article are solely those of the authors and do not necessarily represent those of their affiliated organizations, or those of the publisher, the editors and the reviewers. Any product that may be evaluated in this article, or claim that may be made by its manufacturer, is not guaranteed or endorsed by the publisher.
